# Eleven Monovarietal Extra Virgin Olive Oils from Olives Grown and Processed under the Same Conditions: Effect of the Cultivar on the Chemical Composition and Sensory Traits

**DOI:** 10.3390/foods9070904

**Published:** 2020-07-09

**Authors:** Giuseppe Di Lecce, Maria Piochi, Deborah Pacetti, Natale G. Frega, Edoardo Bartolucci, Serena Scortichini, Dennis Fiorini

**Affiliations:** 1 Independent Researcher, Expert in Food Science and Technology, 26100 Cremona, Italy; leccegius1980@gmail.com; 2 University of Gastronomic Sciences, Piazza Vittorio Emanuele 9, 12042 Pollenzo, Italy; m.piochi@unisg.it; 3 Department of Agricultural, Food, and Environmental Sciences, Polytechnic University of Marche, Via Brecce Bianche, I-60131 Ancona, Italy; n.g.frega@univpm.it (N.G.F.); e.bartolucci@pm.univpm.it (E.B.); 4 School of Science and Technology, Chemistry Division, University of Camerino, V.S. Agostino 1, I-62032 Camerino, Italy; serena.scortichini@unicam.it (S.S.); dennis.fiorini@unicam.it (D.F.)

**Keywords:** monocultivar, autochthonous, cultivar, sensory properties, phenolic substances, triacylglycerols

## Abstract

Eleven Italian monovarietal extra virgin olive oils (MEVOOs) (Carboncella, Coratina, Frantoio, Leccino, Marzio, Maurino, Moraiolo, Piantone di Falerone, Pendolino, Rosciola, Sargano di Fermo) from olives grown in the same experimental olive orchard, under the same conditions (fertilization, irrigation), and processed with the same technology (three-way continuous plant) were investigated. As a result, the impact of the olive cultivar on fatty acid and triacylglycerols composition, oxidative stability, polar phenolic profile and sensory properties (panel test) of the oil was assessed. Pendolino, Maurino and Marzio oils presented the highest levels (*p* < 0.01) of palmitic, linoleic and linolenic acids % and the lowest oleic:linoleic ratio. Within triacylglycerols, triolein (OOO) strongly varied among the oils, with Coratina and Leccino having the highest content. Frantoio showed the lowest 1-Stearoyl-2-palmitoyl-3-oleylglycerol and 1,3-Distearoyl-2-oleylglycerol amounts. Rosciola showed the highest level (*p* < 0.01) for two of the most abundant secoiridoid derivatives (the dialdehydic forms of decarboxymethyl elenolic acid linked to hydroxytyrosol and tyrosol). A good correlation was found between total phenolic content and oxidative stability, indicating Marzio and Leccino respectively as the richest and poorest genotypes. Sensory variability among varieties was mainly linked to perceived bitterness, pungency and fruitiness, while no effects were found on secondary flavors.

## 1. Introduction

Olive oil markets are changing rapidly. Monovarietal extra virgin olive oil (MEVOOs) are gaining increasing interest allowing for further segmenting of the market and creating new trends in high market niches [1,2].

MEVOOs are defined as oils obtained by the transformation of olives from one variety. Traditionally, EVOOs are made of blends of all the olive varieties present on each farm but more recently, milling technology and machinery allow for separate milling of even small quantities of olives [3].

In 2017/2018, Italy was the second largest European olive oil producer after Spain [4] and it is currently the leading country for cultivar biodiversity, accounting for over 800 varieties [5]. Since MEVOOs are products reflecting the characteristics of a country beyond genetics, their systematic sensory and chemical characterization has a pivotal role in order to identify quality oils with remarkable diversity and clear identity. A detailed description of the chemical and sensory traits of MEVOO produced with olive cultivars among the most widespread in Italy, such as Frantoio, Leccino and Moraiolo was reported by several authors [6,7,8,9]. Additionally, the features of MEVOO from cultivars typical of different Italian regions producing appreciated oils were also highlighted [10,11,12,13,14,15,16].

However, a frequent limitation in studies on MEVOOs is that cultivars often come from different geographical areas [14,17,18]; therefore, other variables like the pedoclimatic characteristics may introduce bias in the characterization. In fact, it is known that the same cultivar grown in different pedoclimatic conditions (altitude, latitude, climatic conditions, soil composition etc.) shows different values in fatty acid composition, phenolic content and oxidative stability [19,20,21]. Since pedoclimatic aspects, olive ripeness, harvesting time and the extraction system, strongly impact on the chemical composition and sensory properties of oils [22,23,24], it is recommended to control these factors when studying characteristics of MEVOOs. Within the heritage of Mediterranean diet products, MEVOOs represent precious contributions, whose sensory and healthy properties are explained by chemical compositional peculiarities, in many cases not yet investigated.

Thus, the present study aimed to perform a chemical and sensory characterization of eleven different MEVOOs. Some of the cultivars investigated (Leccino, Frantoio, Maurino, Moraiolo, Pendolino) are well known and widely cultivated in several Italian areas having adequate pedoclimatic conditions, while other cultivars are less diffused and present only in their native regions (Carboncella, Coratina, Marzio, Piantone di Falerone, Rosciola, Sargano di Fermo). Beyond the characterization of MEVOOs from minor cultivars never investigated before, an important outcome of this study is the contribution to understanding the effect that the genetic background of the fruit (effect of cultivar) plays on the chemical composition and sensory properties of the oil. In fact, in the present study all the other parameters are the same for all the cultivars investigated, i.e., olives are grown in the same experimental olive orchard and under the same conditions (fertilization, irrigation) and processed with the same technology.

## 2. Materials and Methods

### 2.1. Standard, Reagents and Solvents

The Folin–Ciocalteu reagent and gallic acid were obtained from Merck & Co. Inc. (Darmstadt, Germany). The fatty acid methyl esters, triacylglycerols, pyridine, 1,1,1,3,3,3-Hexamethylsiloxane and chloroxilane were purchased from Sigma-Aldrich Inc. (St. Louis MO, USA). The phenols *p*-Hydroxyphenylethanol (*p*-HPEA), 3,4-Dihydroxyphenylacetic acid, vanillic acid vanillin, oleuropein, luteolin and apigenin, were purchased from Extrasynthése (Genay, France), Sigma (St. Louis, MO, USA) and Fluka (Buchs, Switzerland). High Pressure Liquid Chromatography (HPLC) grade solvents were purchase from Merck (Darmstadt, Germany). All the solvents and solutions were filtered through a 0.45 μm politetrafluoroetilene filter (PTFE, Supelco, Bellefonte, PA, USA).

### 2.2. Olive Oil Samples

MEVOOs obtained from eleven Italian cultivars were studied. Olive fruits, collected in the crop year 2018/2019, were all provided from the experimental farm “Pasquale Rosati” of the Polytechnic University (Ancona, Italy), where the olive trees were cultivated under identical agronomic and pedoclimatic conditions. However, some of the varieties investigated are currently cultivated on national scale (Leccino, Frantoio, Maurino, Moraiolo, Pendolino), while others are autochthonous of three Italian regions: Marche (Carboncella, Piantone di Falerone, Rosciola, Sargano di Fermo), Tuscany (Marzio) and Apulia (Coratina).

The healthy fruits were harvested by handpicking at the same maturity index (values around 3.5, based on the color and texture of the olive drupe according to the Jaen index [25]: 100 olive fruits were classified into eight different groups and the index was calculated as Σ(A_i_n_i_)/100, where A is the group number and n is the number of fruits in the group). After harvesting, the olive fruits were processed by continuous system technology. For each cultivar, approximately 350–400 kg of olives were collected, and each batch was processed in a three-way continuous plant (P. Barigelli & C., Cingoli, Italy). The olive fruits were defoliated and washed prior to crushing, and then processed by hammer crusher and malaxer. The temperature of the pulp in the malaxer was set at 26 °C. The olive oil was separated by B/D 400 decanter (P. Barigelli & C., Cingoli, Italy) and poured into green sealed glass bottles (0.25 mL each) and the headspace was approximately 10 mL. The EVOO bottles were stored in the dark and at room temperature (20 °C ± 1 °C) and were opened after five months for the analysis. Each analysis was performed in triplicate.

### 2.3. Determination of Legal Quality Parameters

The free acidity (FA, g oleic acid in 100 g of oil), peroxide value (PV, mg eq O_2_ kg^–1^ of oil) and UV spectrophotometric determinations were carried out for each oil sample according to the EEC Reg. no. 2568/1991 and subsequent modifications. Spectrophotometric determinations K_232_, K_270_ and ΔK were carried out using an ultraviolet, visible light and near infrared spectrophotometer (UV-Vis-Nir Cary5000, Varian, Leiní, Italy).

### 2.4. Sensory Evaluation according to the Panel Test

The sensory evaluation was performed by a trained panel (O.L.E.A. Organizzazione Laboratorio Esperti Assaggiatori, Pesaro, Italy) accredited by the Ministry of Agricultural, Food and Forestry Policies (MIPAAF) and according to the procedure reported in the EEC Reg. no. 2568/1991 and in its subsequent modifications. Panelists used a profile sheet adapted from the International Olive Council (IOC) method for designation of origin [26]. The vocabulary included 12 positive attributes: nine descriptors for volatile sensations perceived by retro-olfaction (fruity, greenly fruity, ripely fruity, olive leaf, grass, artichoke, tomato, almond, apple), two tastes (bitter, sweet) and one kinesthetic sensation (pungency). Trained assessors could also mark defects if perceived. Samples (15 mL) were served in standard glass [27] and codified with random three-digit codes. Samples were assessed in three evaluation sessions and served in balanced and randomized order across panelists.

### 2.5. Determination of Fatty Acid Composition

To determine fatty acid composition, fatty acid methyl esters (FAMEs) were obtained with 1 M KOH in methanol [28], and analyzed using a gas chromatograph (GC) HRGC Mega 2 series Model MFC 800 (Fisons Instruments, Milan, Italy). The GC instrument was equipped with a flame ionization detector (FID) and a fused silica capillary column coated with 80% biscyanopropyl/20% cyanopropylphenyl polysiloxane (SP 2330, 60 m length × 0.25 mm i.d. 0.2 m film thickness, Supelco, St. Louis, MO, USA). The carrier gas was helium (2 mL min^–1^); the splitting ratio was 1:80. The injector and detector temperatures were set at 250 °C; the temperature program started at 150 °C and was raised to 220 °C at a rate of 3 °C min^–1^ and was held for 30 min. The FAMEs were identified by comparison with known standards.

### 2.6. Triacylglycerol Determination

A 1 g aliquot of oil was added with internal standard solution (triundecanoin, 1 mg/mL) was silylated according to Sweeley et al. [29] and injected into a GC-FID (HRGC Model 5300, Carlo Erba, Milan, Italy) equipped with a fused-silica capillary column coated with a 50% phenyl-/50% methylpolysiloxane (CP-TAP, 60 m × 25 mm × 0.25 mm i.d., film thickness 0.1 mm, Varian Walnut Creek, CA, USA). The chromatographic method was set according to Boselli et al. [30]. Peak identification was carried out by comparison of the relative retention time with those reported in the literature and with the retention times of the standard substances [31]. Quantitative analyses were performed adopting the corrected area normalization method (with triundecanoin as internal standard).

### 2.7. Determination of the Oxidative Stability

The oxidative stability of the oils was determined by Rancimat apparatus (Metrohm model 679, Herisau, Switzerland), measuring the induction time in response to forced oxidation (induction period) of 5 g sample heated at 110 °C under an air flow of 20 L h^–1^. The induction period (expressed in hours) was determined by drawing the two tangents of the time–conductivity curve and projecting the intersection onto the time-axis.

### 2.8. Phenols Determinations by Folin-Ciocalteu Assay and High-Performance Liquid Chromatography (HPLC) Coupled with Diode Array Detector (DAD)

Phenolic compounds were extracted three times following the procedure described by Boselli et al. [32]. The phenols extracted for Folin–Ciocalteu assay were resuspended in 1 mL methanol and the total phenol content was determined at 765 nm according Singleton et al. [33]. The results were expressed as gallic acid equivalents (mg kg^–1^ oil) based on a calibration curve (*R*^2^ = 0.993). Phenols were also quantified by HPLC coupled with a diode array detector (DAD) and a 3,4-Dihydroxyphenylacetic acid solution was used as internal standard. After the extraction procedure, the dry extracts were resuspended in 1 mL methanol and the solutions were filtered through 0.2 mm regenerated cellulose filters (Schleicher & Schuell, Dassel, Germany). Phenolic compounds were separated by Chromspher C18 (5 ¼ m particle size, 25 cm × 4.6 mm i.d. column, Chrompack Middelburg, Netherlands), using a Varian 9010 ternary pump (Walnut Creek, CA, USA). The sample was injected into a 20 mL loop and the mobile phase flow rate was 0.7 mL min^−1^. The gradient elution was carried out according to Fiori et al. [34]. A Varian Prostar PDA 330 was used as detector to acquire phenolic acids, phenyl ethyl alcohols and secoiridoids at 280 nm, while flavones were detected at 350 nm. The data were acquired using Varian Star 6.3 software. Using their respective standards (*R*^2^ = 0.998, 0.999, 0.996 and 0.998), 3,4-dihydroxyphenylethanol (3,4-DHPEA), *p*-hydroxyphenylethanol (*p*-HPEA), vanillic acid and vanillin, respectively, were quantified. Secoiridoids were quantified with oleuropein (*R*^2^ = 0.999), while luteolin and apigenin were quantified with their standard (*R*^2^ = 0.998, R^2^ = 0.996, respectively). For structural elucidation, the HPLC system was coupled online to an LCQ ion-trap mass spectrometer (Thermoquest, San José, CA, USA) as reported by Boselli et al. [32].

### 2.9. Data Analysis

Oils were firstly classified as extra virgin olive oils by official methods [26]; for all attributes the robust coefficient of variation (%) was lower than 20%. To allow a statistical comparison across the cultivars for the perceived intensity of sensory attributes, two-way ANOVA models were conducted separately on intensity values given to each sensory attribute from panel descriptive data (fixed factors: cultivar, assessors). This statistical approach, that can be exploited when the aim is the valorization and differentiation of oils [35,36] (like in the present case), allows the estimation of the effect of the cultivar on the perceived intensity of the sensations expressed by the F Fisher’s ratio, followed by Tukey’s pairwise test conducted on the mean values (*p* < 0.05). Correlations among variables were tested with the Pearson coefficient (*R*) (*p* < 0.05). A principal component analysis (PCA) was conducted on significant chemical and sensory variables, to exploratorily study the relationships among variables and cultivars. Analyses were conducted with XLStat 2019.1.1, Addinsoft, Boston, MA, USA.

## 3. Results and Discussion

### 3.1. Legal Quality Parameters

Table 1 shows that, considering the parameters FA, PV, K_232_, K_270_ and ΔK, all MEVOOs samples complied with limits required for extra virgin olive oil categorization [37]. FA ranged from 0.22 to 0.39% (g oleic acid per 100 g of oil), much lower than 0.8% set for EVOOs, denoting a good quality and healthy status of olives, which were immediately transformed after harvesting. PV and UV spectrophotometric indices are the two main parameters indicating the oil rancidity progress state. Values for the peroxide and UV indices were lower than the legal limits (PV < 20 meq O_2_ per kg of oil -meq O_2_ kg^–1^-; K_232_ < 2.5, K_270_ < 0.22 and ΔK < 0.01). The low PV levels ranged between 4.0 and 7.2 meq O_2_ kg^–1^, while UV indices did not show significant differences across the samples.

### 3.2. Sensory Evaluation According to the Panel Test

As expected from the optimal quality of olive fruits and the technological practices, no sensory defect was detected. From two-way ANOVA models on the intensity of positive attributes (Table 2), five attributes significantly differed across oils from different varieties, as indicated by the significant Fisher’s F ratio from ANOVA models: fruity (F = 2.1, *p* < 0.03), greenly fruity (F = 2.39, *p* < 0.01), bitter (F = 4.89, *p* < 0.001), sweet (F = 5.52, *p* < 0.001) and pungency (F = 4.14, *p* < 0.001). Secondary descriptors (olive leaf, grass, artichoke, tomato, almond, apple, ripely fruity) were occasionally perceived but at low intensities (≤2.5) and without significant differences (*p* > 0.05) among the oils. The lack of significant differences across varieties did not allow a clear diversification, probably due to the low intensity values of these secondary descriptors. Other reports similarly showed that secondary notes differ slightly across cultivars [5,38] as compared to major attributes, e.g., bitterness and pungency, and they are perceived at modest/low intensities.

As an example, Cantini et al. [38] reported a maximum intensity of 3.0 for secondary notes such as artichoke or almond in 57 cultivars investigated. Instead, bitter, pungency and greenly fruity seemed more related to the cultivar and less linked to agronomical and pedoclimatic influences [39].

Marzio MEVOO was characterized by the significantly highest greenly fruity, bitterness and pungency. Moraiolo was also characterized by a high fruity and greenly fruity (the highest), with a pronounced pungency and bitterness balanced by the sweetness. Leccino was the sweetest, and had the significantly lowest greenly fruity, bitterness and pungency intensities. These results are in agreement with previous reports, describing Coratina as significantly more bitter than Leccino [5]. Sensory similarities were found with some attributes that did not significantly differ across cultivars, such as the intensity of pungency, similar in Marzio, Sargano di Fermo, Rosciola, Piantone di Falerone, Maurino, Frantoio, Moraiolo and Pendolino and bitterness, at the same intensity in Marzio, Coratina, Rosciola, Pendolino, Moraiolo, Carboncella and Sargano di Fermo.

### 3.3. Fatty Acid Composition

The composition of the principal fatty acids of MEVOOs is shown in Table 3. In all the MEVOOs, fatty acids percentages were compliant with the legal limits imposed by EEC Reg. no. 2568, 1991. Moreover, the investigated samples showed fatty acid compositions that are well in the average value ranges reported in literature for various Italian monovarietal oils [11,12,40].

On the whole, our findings corroborated the hypothesis that the oil fatty acid profile is strongly under genetic control. Ben Ayed et al. [41] noticed that oleic acid amounts in olive oil can be strongly related to the polymorphisms of fatty acid-related genes, such as the stearoyl-acyl carrier protein desaturase gene (SAD). TT-SAD.1 genotype was found to be associated with a higher proportion of monounsaturated fatty acids, mainly oleic acid, as well as with lower proportions of palmitic acid, thus causing olive varieties with this genotype to produce more monounsaturated fatty acids, namely oleic acid, than saturated fatty acids. 

Indeed, we found clear differences among the MEVOO’s fatty acid compositions, mainly in terms of palmitic, oleic, linoleic and linolenic acid contents. Since the investigated oils were obtained with olives cultivated in the same growing conditions and were processed with the same operative conditions, the highlighted differences were related to the olive cultivar.

In detail, the oleic acid content, ranging from 71.55 to 78.42%, clustered the oils into two groups: the first composed of eight varieties (Coratina, Rosciola, Sargano di Fermo, Frantoio, Carboncella, Moraiolo, Leccino, Piantone di Falerone), with the significantly highest values (*p* < 0.001), ranging from 76.2 to 78.4%, and the second with Pendolino, Maurino and Marzio oils, with the lowest values (72.0–73.0%). Simultaneously, Pendolino, Maurino and Marzio MEVOOs presented the significantly highest levels of palmitic, linoleic and linolenic acids. Thus, Pendolino, Maurino and Marzio oils stood out from the rest for their unfavorable oxidative stability parameters, such as the highest unsaturation index and lowest oleic:linoleic ratio. It is important to notice that high unsaturation index, low percent content of oleic acid and high percent content of linoleic acid bound in the acylglycerol backbone, make olive oil weakly resistant toward oxidation.

Our findings were in line with previous studies reporting the comparison of fatty acid composition in some of the MEVOOs investigated by us, e.g., Bianchi et al. [36] studied the fatty acid profile of Frantoio, Coratina and Moraiolo oils from olives harvested in different Italian regions (i.e., Apulia, Tuscany) revealing that these oils were similar on the basis of oleic acid content. Blasi et al. [6] did not underline significant differences, in terms of overall fatty acids composition, among Frantoio, Leccino and Moraiolo MEVOOs purchased from producers located in central Italy regions.

However, the relative fatty acid composition found in the different MEVOOs is different as compared to that reported by Portarena et al. for the same varieties [7]. They also investigated MEVOOs processed with the same plant and obtained from olives cultivated in the same area (Perugia, Italy). Differently from our results, they found that Moraiolo differed from Frantoio and Leccino oils in terms of oleic and linoleic acids percentages. These different outcomes can be related to the different environmental conditions thus suggesting a synergistic effect of genetic and environmental factors on the fatty acid composition. Mousavi et al. [20] investigated several olive cultivars, including Frantoio, Leccino, Coratina and Moraiolo, and demonstrated that the fatty acid profile of the oil was regulated by the interaction of environmental and genotype factors. It has been shown that both temperature and light play a role in modulating oleic acid content and the oleic acid/(palmitic + linoleic acids) ratio in the oil.

### 3.4. Triacylglycerol Composition

Although triacylglycerols (TAGs) have been widely utilized as markers of varietal and geographical origin of EVOO [42,43,44,45,46,47], the investigation of TAG profiles in Italian MEVOO is still limited [6,21,48,49,50]. To the best of our knowledge, the present work represents the first attempt at characterization of the TAGs profile in Italian MEVOO from Carboncella, Marzio, Maurino, Piantone di Falerone and Sargano di Fermo cultivars.

Significant variations in terms of TAG composition (Table 3) were found across the samples.

In all the oils, the most abundant TAG was OOO, followed by POO and OOL. They made up 80–90% of the total TAG profile. The remaining part of TAG matter was mainly formed of PPO, POL and SOO species, which accounted for about 10–15% of TAG profile.

Considering the most abundant TAGs, OOO ranged from 35.0 ± 2.9 to 42.4 ± 3.1%, POO from 26.9 ± 1.9 to 34.7 ± 2.2%, OOL from 10.0 ± 1.9 to 13.8 ± 1.9%. These results are comparable to those reported for some Italian monovarietal olive oils, including Coratina, Leccino and Pendolino [48,50].

The POO and OOL levels weakly changed across the cultivars. Coratina oil presented the significant highest POO level (34.7 ± 2.2%), Marzio and Maurino the lowest one (26.9 ± 1.9% and 29.8 ± 3.2%, respectively), the remaining oils had comparable POO amounts accounting for about 30.6–32.8%. Similarly, all the samples presented comparable OOL content (from 10.0 to 13.8%), except for Moraiolo oil which had significantly higher levels (15.7 ± 2.0%) than all the other samples.

Unlike POO and OOL, OOO levels strongly varied among the oils. Although Marzio, Maurino and Pendolino showed similar OOO amounts, only Marzio and Maurino differed from all the other samples. They presented significantly (*p* < 0.001) lowest OOO levels. Conversely, Pendolino was not different form all the other samples, except than Coratina and Leccino, that presented the highest OOO levels. Our results are in good agreement with those of Giuffrè [48] who found higher OOO levels in Coratina than in Pendolino oils.

Congruently to what was observed for oleic acid content, the OOO level enables the discrimination of Marzio and Maurino oils from all the others. Anyway, on the basis of OOO levels it was not possible to differentiate Pendolino from the other oils. These outcomes lead us to suppose that the investigation of TAG profiles provides more restrictive information on oil discrimination than that derived from analysis of total fatty acid profiles. In fact, variation of TAG profiles among MEVOOs could better reflect the specific metabolic behavior of each cultivar. The biosynthesis of TAG in the olive fruit involves additional pathways with respect to the biosynthesis of fatty acids. This assumption can also be reinforced by considering the variation of TAG species formed by the combination of oleic acid and the most abundant saturated fatty acids, such as palmitic and stearic acids. Although stearic acid clustered the oils into two groups (Carboncella, Coratina, Marzio, Moraiolo, Rosciola vs. the other MEVOOs), the level of the main molecular species containing stearic, SOO and POS, enabled the discrimination of Frantoio oil from all the others, since Frantoio oil showed the lowest SOO and POS amounts. Similarly, although the highest levels of palmitic and the lowest level of oleic acids distinguished Marzio, Maurino and Pendolino oils, the highest PPO level differentiated Marzio and Moraiolo from the rest of the oils.

### 3.5. Oxidative Stability, Total Phenols Content and Phenolic Profile

Table 4 reports the oxidative stability, total phenolic content and the amount of the single phenolic compounds identified in the oil samples.

The induction time of the oils ranged from 17.5 (Leccino) to 29.5 (Coratina) hours. Coratina and Rosciola showed the significantly highest stability.

The total amount of phenols determined by Folin–Ciocalteu assay ranged from 153 to 396 mg kg^–1^. Marzio oil showed the significantly highest content of phenols (396 mg kg^–1^) followed by Carboncella (323.1 mg kg^–1^) and Pendolino (307.6 mg kg^–1^). Leccino showed the significantly lowest amount of phenols (153 mg kg^–1^), while Rosciola, Coratina and Maurino oils showed comparable phenolic content. The total phenol content values agree with the ones reported by other studies conducted on MEVOOs. Baiano et al. [51] reported values between 133 and 322 mg kg^–1^ for olive oils from orchards located in the north of Apulia region, Negro et al. [13] presented values between 138 and 278 mg kg^–1^ for oils produced in the province of Lecce (Apulia, Italy), whereas Ninfali et al. [52] reported values in the range of 50–236 mg kg^–1^ for plants cultivated in the center of Italy. Ricciutelli et al. [53] indicated values ranging from 136 to 437 mg kg^–1^ for commercial EVOOs. Rotondi et al. [5] reported mean values for total phenolic content ranging from 327 to 646 mg kg^–1^ (obtained for the cultivars Biancolilla and Bianchera, respectively) in 16 Italian cultivars considered more representative. However, it is to be reminded that the total phenol content is strictly related to many factors, such as the olive harvesting time, oil extraction techniques or quantification methodologies [54]. Many studies, indeed, showed that the pedoclimatic and technological aspects are the main parameters influencing the total phenol content in EVOOs [9,55,56]. The genotype may also highly influence the oil phenolic content; Negro et al. [13] indicated that genotype may be responsible for about 50%. The phenolic content is usually related to the shelf-life and the oxidative stability of olive oil, although polar phenolic substances are also responsible for the olive oil flavor related to bitterness, astringency and pungency. Bitterness in olive oil is strictly due to the content of oleuropein glucoside and its aglycon [23]. Oils obtained from olive fruits rich in phenolics, e.g., Marzio MEVOO, are expected to be more bitter and pungent than the others. The EU legislation about the health claim on olive oil phenolic substances requires accurate measurements of the level of specific phenolic compounds in olive oil. In the current paper, twelve phenolic compounds were also specifically identified and quantified using HPLC coupled to DAD and a mass spectrometry instrument. The main phenolic alcohols found in the MEVOOs were 3,4-DHPEA and *p*-HPEA. Their concentration is usually low in the fresh oils, but increases during storage [32,57,58,59] due to the lysis of the secoiridoids, such as the dialdehydic forms of decarboxymethyl elenolic acid linked to 3,4-DHPEA (3,4-DHPEA-EDA, oleacein) and to *p*-HPEA (*p*-HPEA-EDA, oleocanthal), and oleuropein aglycon (3,4-DHPEA-EA) that release 3,4-DHPEA and *p*-HPEA, respectively. As shown in Table 4, the content of 3,4-DHPEA ranged between 1.8 to 18.5 mg kg^–1^ for Pendolino and Leccino, respectively, while *p*-HPEA ranged between 2.2 and 16.8 mg kg^–1^ for Moraiolo and Rosciola. Other studies conducted on Leccino MEVOOs reported values of 13.8 mg kg^–1^ [15] and 0.72–1.37 mg kg^–1^ [57] for 3,4-DHPEA content and 20.2 mg kg^–1^ [15] and from 1.08 to 2.22 mg kg^–1^ [60] for *p*-HPEA, indicating a certain variability for these phenolics. Ricciutelli et al. [53] have identified a mean value of 9.9 mg kg^–1^ oil and 13.4 mg kg^–1^ oil for 3,4-DHPEA and *p*-HPEA, respectively, in commercial EVOOs. Among phenolic alcohols, 3,4-DHPEA is worthy of investigation for its nutraceutical properties [61], so the cultivars that showed the highest contents (mainly Leccino, Rosciola and Carboncella) are worthy of interest to obtain EVOO blends with increased nutraceutical properties. Thus 3,4-DHPEA has been indicated by Carrasco-Pancorbo et al. [62] as the main contributor among polyphenolic compounds for oxidative stability of olive oils. Vanillic acid and vanillin were found at very low concentrations in all samples (0.2–1.34 mg kg^–1^ and 0.50–3.2 mg kg^–1^ of oil, respectively), with small differences among studied oils. Only in Moraiolo and Piantone di Falerone was vanillic acid not detected. These values are in accordance with the ones found by Gambacorta et al. [60] in MEVOOs investigated (including Coratina, Frantoio and Leccino varieties) and also Ricciutelli et al. [53] indicated an average value for vanillic acid of 0.3 mg kg^–1^ for commercial EVOOs. The secoiridoid compounds are in general the most abundant phenolic compounds present in fresh oils but during shelf life their content decreases [32]. Rosciola showed the highest content for 3,4-DHPEA-EDA and *p*-HPEA-EDA (120 and 105 mg kg^–1^, respectively). Other varieties such as Carboncella, Coratina, Moraiolo and Piantone di Falerone showed a good content of both the phenols compared to the others. Coratina, one of variety appreciated for the high phenols content, showed 103 and 64.4 mg kg^–1^ of 3,4-DHPEA-EDA and *p*-HPEA-EDA, respectively. Frantoio, Marzio, Maurino, Pendolino and Sargano di Fermo cultivars showed an average content of these two compounds compared to Coratina, while Leccino presented the lowest content (21 and 16.7 mg kg^–1^ for 3,4-DHPEA-EDA and *p*-HPEA-EDA, respectively). *p*-HPEA-EDA deserves great attention because of its several nutraceutical properties reported by many studies and reviews [63,64]; it showed wide concentration ranges in olive oils. Backhouche et al. [65] reported values from 3.3 to 4.6 mg kg^–1^ depending on the geographical region, for the Spanish Arbequina variety, while much higher values (104.0 ± 1.8 mg kg^–1^) were reported for the same variety by Vidal et al., in a study aimed to obtain oils rich in oleacein and oleocanthal [66]. This big difference in concentration values can also be explained largely with the different methods and reference standards used to quantify secoiridoid derivatives. Backhouche et al. [65] used electrospray ionization with time of flight mass spectrometer detection and oleuropein as calibration standard, that gave a very different response than tyrosol [65], that instead was used as an external standard by Vidal et al. with an ultraviolet detection at 280 nm [66]. Concentrations from 38.7 to 72.5 mg kg^–1^, depending on the different processing conditions, were reported for the Spanish Picual variety [67]. Fuentes et al. [68] reported concentrations from 25 to 77 mg kg^–1^ for Chilean oils, Negro et al. [13] instead indicated values from 4.3 to 103.4 mg kg^–1^ for Apulian varieties. Considering the important role of *p*-HPEA-EDA in the nutraceutical properties of EVOO, Rosciola and Moraiolo genotypes represent the best sources among varieties investigated, with concentrations of 105 ± 6.9 and 82.3 ± 6.8 mg kg^–1^, respectively.

In all varieties, the dialdehydic form of ligstroside aglycon (DLA) was coeluted with the oxidized form of *p*-HPEA-EDA and the highest content was found in Marzio (147 mg kg^–1^) followed by Pendolino and Sargano di Fermo (126 and 101 mg kg^–1^, respectively), while Leccino and Coratina showed lower content (49.3 and 51.2 mg kg^–1^, respectively). The last two secoiridoids in terms of elution time were 3,4-DHPEA-EA and *p*-HPEA-EA found in all varieties. The contents of 3,4-DHPEA-EA was higher in Marzio (106 mg kg^–1^), followed by Pendolino (72.5 mg kg^–1^), Maurino (57.1 mg kg^–1^) and Carboncella (53.5 mg kg^–1^), while the Leccino variety showed the lowest (3.8 mg kg^–1^). For some varieties Negro et al. [13] reported higher values, in the range of 33.8–152.3 mg kg^–1^, while similar values were reported in the oils analyzed by Ragusa et al. [15]. Normally 3,4-DHPEA-EA tends to decrease from drupes to malaxation paste and to the final oil [13]. *p*-HPEA-EA was the last secoiridoid quantified, its content ranged between 2.9 and 23.3 mg kg^–1^, for Leccino and Marzio, respectively. In this case *p*-HPEA-EA content is similar to the values reported by Negro et al. [13] and slightly lower than the ones reported by Ragusa et al. [15]. The flavonoids that can usually be found in EVOO extracts are luteolin, apigenin and sometimes methoxyluteolin. This class of compounds is known to have many beneficial biological effects including anti-inflammatory, antioxidant and estrogenic activity [69]. Methoxyluteolin was found in traces only in the Moraiolo variety, while luteolin ranged between 2.65 and 8.3 mg kg^–1^ for Piantone di Falerone and Carboncella, respectively. Similar values were found by García-Martínez et al. [69] in Spanish EVOOs (1.66-6.21 mg kg^–1^) and by Tuberoso et al. [66] in varieties from Sardinia region (Italy) (0.2–7.1 mg kg^–1^). Luteolin was not detected in Leccino and Maurino varieties. Finally, apigenin was found with an average content of about 2 mg kg^–1^, in accordance with the values reported for EVOOs in several other studies [60,69,70]. Marzio and Rosciola MEVOOs complied with the minimum content of the specific phenolic substances required to acknowledge the health claim (250 mg kg^–1^) [71].

### 3.6. Sensory Properties of Oils and Relationship between Sensory Sensations and Chemical Composition

**Figure 1:**
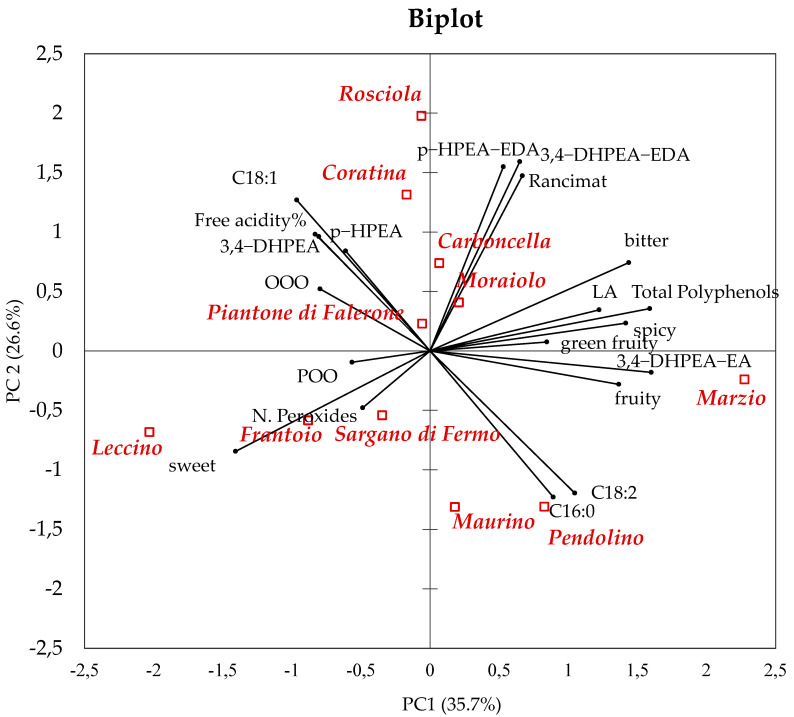
Bi-plot from principal component analysis (PCA) reporting principal components 1 and 2 (PC1 and PC2, respectively) with the loadings of selected chemical and sensory variables and the scores (oil samples).

The bi-plot from PCA illustrates the mutual relationships between samples and discriminating chemical and sensory variables (Figure 1).

Legend: 3,4-DHPEA: 3,4-Dihydroxyphenylethanol; *p*-HPEA: *p*-Hydroxyphenylethanol; 3,4-DHPEA-EDA: dialdehydic form of decarboxymethyl elenolic acid linked to 3,4-DHPEA; *p*-HPEA-EDA: dialdehydic form of decarboxymethyl elenolic acid linked to *p*-HPEA; 3,4-DHPEA-EA: oleuropein aglycon; *p*-HPEA-EA: ligstroside aglycon; C16:0: palmitic acid; C18:1: oleic acid; C18:2: linoleic acid; total phenols (determined by Folin–Ciocalteu method); OOO: 1,2,3-Trioleylglycerol; POO: 2,3-Dioleyl-1-palmitoylglycerol.

The first two components in the PCA accounted for 62.3% of total variance, with the first component (PC1) explaining 35.7%.

Samples were distributed on the PC1 according to a contraposition between bitter, greenly fruity and spicy (positively correlated on PC1) and sweet (negatively correlated on PC1). Along PC1, bitter, greenly fruity and pungency showed a high correlation with total phenols compounds, *p*-HPEA-EA and with the amount of 3,4-DHPEA-EA. This is in agreement with previous studies clearly showing that 3,4-DHPEA-EA is crucial for the perception of bitter and pungency in EVOOs [72]. The positive correlation observed on the bi-plot between oxidative stability and total phenols has been previously documented [32].

Instead, sweet was positively correlated with peroxide index and POO; this finding may be explained by the lower phenols content that protect against the oxidation phenomena and that contribute to pungency/bitterness, attributes lacking in sweet oils. As the correlation on PC2 positively increased, both the oxidative stability and the amount of 3,4-DHPEA-EDA and *p*-HPEA-EDA increased. Acidity, amount of 3,4-DHPEA, *p*-HPEA and oleic acid (C18:1)%, had high positive loadings on PC2.

The content of *p*-HPEA and 3,4-DHPEA, that is known to increase with oil aging, strongly characterized Coratina; these phenols correlated with FA, that in fact also derives from hydrolytic processes.

On PC2, palmitic acid (C16:0) and linoleic acid (C18:2) had negative loadings as well as PV and POO (despite lower loadings).

## 4. Conclusions

In the present paper, a chemical and sensory characterization was conducted on eleven MEVOOs from olives grown in the same experimental olive orchard, with the same conditions (fertilization, irrigation), and processed with the same technology. Thus, differences found across MEVOOs were attributable only to the factors related to the genetic background of the olive cultivar.

The findings highlighted the impact of genetic background of the olive on the sensory attributes, fatty acid, TAG and phenolic compositions of the oils.

Across the investigated oils, Marzio stood out significantly from the rest resulting in the most bitter, pungent, fruity and the richest in phenolic compounds. The high phenolic level conferred it a good oxidative stability although it presented the highest unsaturation index.

The study represents a scientific contribution enriching the database of Italian olive oil cultivars providing information on the role of the cultivar in the differentiation of the chemical composition of the different MEVOOs, by excluding bias associated to the pedoclimatic influence and technological production conditions.

## Figures and Tables

**Table 1:** Olive oil quality parameters of the eleven monovarietal oils investigated.

Cultivar	Free Acidity ^X^	Peroxide Value ^Z^	K_232_	K_270_	∆K
Carboncella	0.33 ^abcd^ ± 0.03	5.10 ^d^ ± 0.07	1.73 ^a^ ± 0.13	0.12 ^a^ ± 0.002	0.003 ^a^ ± 0.001
Coratina	0.34 ^abc^ ± 0.02	6.20 ^bc^ ± 0.14	1.63 ^a^ ± 0.12	0.13 ^a^ ± 0.003	0.002 ^a^ ± 0.001
Frantoio	0.23 ^defg^ ± 0.01	6.27 ^b^ ± 0.10	1.59 ^a^ ± 0.11	0.10 ^a^ ± 0.002	0.003 ^a^ ± 0.001
Leccino	0.32 ^bcde^ ± 0.03	7.15 ^a^ ± 0.07	1.83 ^a^ ± 0.19	0.15 ^a^ ± 0.005	0.002 ^a^ ± 0.001
Marzio	0.25 ^g^ ± 0.02	5.96 ^bc^ ± 0.08	1.86 ^a^ ± 0.16	0.14 ^a^ ± 0.004	0.005 ^a^ ± 0.001
Maurino	0.28 ^cdef^ ± 0.01	5.05 ^d^ ± 0.07	1.77 ^a^ ± 0.11	0.16 ^a^ ± 0.006	0.003 ^a^ ± 0.001
Moraiolo	0.21 ^fg^ ± 0.01	4.12 ^e^ ± 0.16	1.65 ^a^ ± 0.19	0.13 ^a^ ± 0.004	0.003 ^a^ ± 0.001
Piantone di Falerone	0.39 ^a^ ± 0.04	6.80 ^a^ ± 0.15	1.73 ^a^ ± 0.24	0.11 ^a^ ± 0.002	0.003 ^a^ ± 0.001
Pendolino	0.22 ^efg^ ± 0.02	6.85 ^a^ ± 0.10	1.76 ^a^ ± 0.18	0.15 ^a^ ± 0.003	0.004 ^a^ ± 0.001
Rosciola	0.38 ^ab^ ± 0.04	5.90 ^c^ ± 0.12	1.76 ^a^ ± 0.17	0.13 ^a^ ± 0.004	0.002 ^a^ ± 0.001
Sargano di Fermo	0.28 ^cdef^ ± 0.02	6.85 ^a^ ± 0.08	1.64 ^a^ ± 0.21	0.10 ^a^ ± 0.001	0.002 ^a^ ± 0.001

Results are expressed as mean ± standard deviation (*n* = 3); ^x^ g oleic acid in 100 g of oil; ^z^ mg eq O_2_ kg^–1^ of oil; K_232,_ K_270_: UV absorption at ʎ = 232 and 270 nm; different letters in the same column indicate significantly different values (*p* < 0.001).

**Table 2:** Sensory evaluation and perceived intensity (expressed as means of ratings given by assessors ± standard error of the means) of the main sensory attributes in the investigated monovarietal oils.

Cultivar	Fruity	Bitter	Pungency	Greenly Fruity	Sweet
Carboncella	2.6 ^ab^ ± 0.2	3.0 ^abc^ ± 0.3	2.9 ^bc^ ± 0.2	1.7 ^b^ ± 0.4	2.4 ^c^ ± 0.3
Coratina	3.1 ^ab^ ± 0.2	3.6 ^ab^ ± 0.3	3.1 ^abc^ ± 0.2	1.9 ^ab^ ± 0.4	2.7 ^bc^ ± 0.3
Frantoio	2.9 ^ab^ ± 0.2	1.9 ^c^± 0.3	3.1 ^abc^ ± 0.2	2.0 ^ab^ ± 0.4	4.0 ^ab^ ± 0.3
Leccino	2.5 ^b^ ± 0.2	1.9 ^c^ ± 0.3	2.1^c^ ± 0.2	1.8 ^ab^ ± 0.4	4.5 ^a^± 0.3
Marzio	3.5 ^a^ ± 0.2	4.1 ^a^ ± 0.3	4.1 ^a^ ± 0.2	3.2 ^ab^ ± 0.4	2.3 ^c^ ± 0.3
Maurino	3.1 ^ab^ ± 0.2	2.3 ^bc^ ± 0.3	3.2 ^abc^ ± 0.2	2.2 ^ab^ ± 0.4	3.5 ^abc^ ± 0.3
Moraiolo	3.3 ^ab^ ± 0.2	3.0 ^abc^ ± 0.3	3.1 ^abc^ ± 0.2	3.8 ^a^ ± 0.4	3.4 ^abc^ ± 0.3
Piantone di Falerone	3.3 ^ab^ ± 0.2	3.0 ^abc^ ± 0.3	3.2 ^abc^ ± 0.2	2.5 ^ab^ ± 0.4	2.9 ^bc^ ± 0.3
Pendolino	3.1 ^ab^ ± 0.2	2.7 ^bc^ ± 0.3	3.1^abc^ ± 0.2	2.5 ^ab^ ± 0.4	2.9 ^bc^ ± 0.3
Rosciola	2.9 ^ab^ ± 0.2	3.2 ^abc^ ± 0.3	3.5 ^ab^ ± 0.2	2.8 ^ab^ ± 0.4	2.7 ^bc^ ± 0.3
Sargano di Fermo	3.2 ^ab^ ± 0.2	3.0 ^abc^ ± 0.3	3.6 ^ab^ ± 0.2	3.2 ^ab^ ± 0.4	3.8 ^ab^ ± 0.3

Different letters in the same column indicate significantly different values: fruity (*p* < 0.03), greenly fruity (*p* < 0.01), bitter (*p* < 0.001), sweet (*p* < 0.001) and pungency (*p* < 0.001) obtained from two-way ANOVA models (fixed factors: cultivar, assessors), followed by Tukey’s pairwise test (*p* < 0.05).

**Table 3:** Fatty acid composition, oleic acid:linoleic acid ratio, insaturation index and triacylglycerols composition of the eleven monovarietal extra virgin olive oils investigated.

Compounds	Carboncella	Coratina	Frantoio	Leccino	Marzio	Maurino	Moraiolo	Piantone di Falerone	Pendolino	Rosciola	Sargano di Fermo
Fatty acids (%)											
Palmitic acid	13.1 ^c^ ± 0.6	13.6 ^c^ ± 0.6	13.4 ^c^ ± 0.7	13.7 ^bc^ ± 0.7	14.6 ^a^ ± 0.4	15.1 ^a^ ± 0.8	13.5 ^bc^ ± 0.5	13.9 ^bc^ ± 0.5	15.3 ^a^ ± 0.8	12.9 ^c^ ± 0.8	13.3 ^bc^ ± 0.8
Palmitoleic acid	0.98 ^b^ ± 0.1	1.17 ^a^ ± 0.3	1.08 ^a^ ± 0.2	1.26 ^a^ ± 0.2	0.81 ^b^ ± 0.1	1.26 ^a^ ± 0.1	0.94 ^b^ ± 0.1	1.12 ^a^ ± 0.1	1.10 ^a^ ± 0.2	0.97 ^b^ ± 0.1	1.04 ^ab^ ± 0.1
Stearic acid	1.97 ^a^ ± 0.2	1.91 ^a^ ± 0.3	1.83 ^b^ ± 0.4	1.69 ^b^ ± 0.2	1.92 ^a^ ± 0.3	1.68 ^b^ ± 0.2	1.89 ^a^ ± 0.2	1.94 ^a^ ± 0.1	1.71 ^b^ ± 0.1	2.03 ^a^ ± 0.2	1.85 ^b^ ± 0.2
Oleic acid	77.1 ^a^ ± 4.2	78.8 ^a^ ± 3.9	76.7 ^a^ ± 4.3	76.7 ^a^ ± 4.1	73.0 ^bc^ ± 3.8	72.4 ^bc^ ± 3.5	77.6 ^a^ ± 4.1	75.7 ^ab^ ± 3.7	72.0 ^c^ ± 3.8	77.8 ^a^ ± 4.5	76.1 ^a^ ± 4.1
Linoleic acid	7.21 ^b^ ± 0.7	4.82 ^f^ ± 0.3	6.21 ^cd^ ± 0.5	5.95 ^de^ ± 0.4	8.80 ^a^ ± 0.7	8.72 ^a^ ± 0.6	5.41 ^e^ ± 0.5	6.62 ^bc^ ± 0.5	8.95 ^a^ ± 0.6	5.62 ^e^ ± 0.4	6.93 ^bc^ ± 0.4
Linolenic acid	0.62 ^c^ ± 0.1	0.64 ^c^ ± 0.1	0.71 ^c^ ± 0.1	0.72 ^c^ ± 0.1	0.81 ^ab^ ± 0.1	0.83 ^ab^ ± 0.1	0.70 ^c^ ± 0.2	0.62 ^c^ ± 0.1	0.95 ^a^ ± 0.1	0.63 ^c^ ± 0.1	0.62 ^c^ ± 0.1
Oleic acid/linoleic acid	10.6 ^c^ ± 0.8	16.3 ^a^ ± 1.1	12.3 ^bc^ ± 0.9	12.9 ^bc^ ± 0.8	8.25 ^d^ ± 0.5	8.30 ^d^ ± 0.6	14.5 ^b^ ± 1.0	11.4 ^bc^ ± 0.9	8.08 ^d^ ± 1.1	13.9 ^b^ ± 0.8	10.9 ^c^ ± 0.8
Insaturation index^z^	161.1 ^b^ ± 11	139.6 ^cd^ ± 9	151.6 ^c^ ± 11	151.6 ^c^ ± 12	178.6 ^a^ ± 13	177.4 ^a^ ± 12	146.2 ^c^ ± 9.7	155.5 ^c^ ± 10	181.2 ^a^ ± 15	147.1 ^c^ ± 9.8	161.2 ^b^ ± 12
**Triacylglycerols (%)**											
PPO	5.6 ^b^ ± 0.5	3.0 ^c^ ± 0.4	2.9 ^c^ ± 0.4	6.0 ^ab^ ± 0.5	7.1 ^a^ ± 0.5	5.6 ^b^ ± 0.5	7.1 ^a^ ± 0.7	3.3 ^c^ ± 0.5	3.4 ^c^ ± 0.4	4.2 ^c^ ± 0.6	5.1 ^b^ ± 0.4
PPL + OPPo	2.1 ± 0.3	2.5 ± 0.3	1.8 ± 0.4	3.1 ± 0.2	2.3 ± 0.4	2.3 ± 0.4	2.9 ± 0.5	1.9 ± 0.3	2.1 ± 0.4	2.2 ± 0.5	2.0 ± 0.3
POS	2.2 ^a^ ± 0.4	2.1 ^a^ ± 0.3	1.6 ^b^ ± 0.3	2.6 ^a^ ± 0.3	2.0 ^a^ ± 0.2	2.9 ^a^ ± 0.5	2.3 ^a^ ± 0.6	2.3 ^a^ ± 0.4	2.7 ^a^ ± 0.5	2.3 ^a^ ± 0.3	2.2 ^a^ ± 0.4
POO	32.8 ^ab^ ± 2.3	34.7 ^a^ ± 2.2	33.6 ^ab^ ± 2.1	32.3 ^ab^ ± 1.8	26.9 ^b^ ± 1.9	29.8 ^b^ ± 3.2	32.4 ^ab^ ± 2.6	30.6 ^ab^ ± 2.9	30.8 ^ab^ ± 3.5	32.2 ^ab^ ± 2.71	32.6 ^ab^ ± 2.3
POL + OOPo	6.5 ^bc^ ± 0.9	4.8 ^c^ ± 0.5	5.3 ^c^ ± 0.8	7.9 ^ab^ ± 0.8	8.9 ^a^ ± 0.7	6.2 ^c^ ± 0.8	9.5 ^a^ ± 0.6	4.3 ^c^ ± 0.9	5.6 ^c^ ± 0.5	5.2 ^c^ ± 0.4	5.2 ^c^ ± 0.9
PLL + PoOL	0.6 ^b^ ± 0.2	0.5 ^b^ ± 0.3	0.4 ^b^ ± 0.1	0.3 ^b^ ± 0.1	0.5 ^b^ ± 0.2	0.3 ^b^ ± 0.1	0.9 ^a^ ± 0.2	0.4 ^b^ ± 0.1	0.5 ^b^ ± 0.2	0.3 ^b^ ± 0.1	0.3 ^b^ ± 0.1
SSO	0.2 ± 0.0	0.4 ± 0.1	0.3 ± 0.1	0.3 ± 0.1	0.3 ± 0.1	0.4 ± 0.1	0.5 ± 0.2	0.3 ± 0.1	0.4 ± 0.1	0.4 ± 0.1	0.3 ± 0.1
SOO	4.9 ^b^ ± 0.6	4.4 ^b^ ± 0.7	2.8 ^c^ ± 0.9	5.4 ^b^ ± 0.8	4.2 ^b^ ± 0.8	4.7 ^b^ ± 0.6	4.3 ^b^ ± 0.8	4.9 ^b^ ± 1.1	6.0 ^a^ ± 0.6	5.2 ^b^ ± 0.7	4.9 ^b^ ± 0.8
OOO	40.3 ^ab^ ± 1.8	42.2 ^a^ ± 2.0	41.4 ^a^ ± 3.1	39.0 ^ab^ ± 2.2	35.0 ^c^ ± 2.9	36.3 ^c^ ± 3.4	39.4 ^ab^ ± 3.1	39.0 ^ab^ ± 2.7	37.5 ^bc^ ± 2.7	42.4 ^a^ ± 3.1	39.3 ^ab^ ± 2.8
OOL	11.6 ^b^ ± 1.7	11.8 ^b^ ± 2.0	10.3 ^b^ ± 1.8	13.8 ^b^ ± 1.9	12.3 ^b^ ± 2.3	11.7 ^b^ ± 2.5	15.7 ^a^ ± 2.0	10.0 ^b^ ± 1.9	12.8 ^b^ ± 2.3	11.7 ^b^ ± 2.1	10.7 ^b^ ± 1.8
OLL	0.2 ± 0.0	0.2 ± 0.0	0.1 ± 0.0	0.2 ± 0.0	0.6 ± 0.2	0.2 ± 0.0	0.4 ± 0.1	0.2 ± 0.0	0.1 ± 0.0	0.2 ± 0.1	0.2 ± 0.1
LLL	0.6 ^a^ ± 0.2	0.7 ^a^ ± 0.2	0.4 ^ab^ ± 0.2	0.6 ^a^ ± 0.1	0.5 ^a^ ± 0.2	0.5 ^a^ ± 0.2	0.5 ^a^ ± 0.2	0.3 ^b^ ± 0.1	0.5 ^a^ ± 0.2	0.5 ^a^ ± 0.2	0.4 ^a^ ± 0.1
AOO	0.4 ± 0.1	0.4 ± 0.1	0.3 ± 0.1	0.5 ± 0.2	0.4 ± 0.1	0.5 ± 0.2	0.3 ± 0.1	0.3 ± 0.1	0.4 ± 0.2	0.3 ± 0.1	0.3 ± 0.1

Results expressed as g of fatty acid methyl ester 100 g^–1^ of oil; different letters in the same row indicate significantly different values (*p* < 0.001); ^z^ Calculated as Σ (% monounsaturated + (diunsaturated × 10) + (triunsaturated × 20)) /100; Triacylglycerols molecular species abbreviations: 1,2-Dipalmitoyl-3-oleylglycerol (PPO); 1,2-Dipalmitoyl-3-linoleylglycerol (PPL); 1-Oleyl-2-palmitoyl-3-palmitoleylglycerol (OPPo); 1-Stearoyl-2-palmitoyl-3-oleylglycerol (POS); 2,3-Dioleyl-1-palmitoylglycerol (POO); palmitoyl-2-Oleyl-3-linoleylglycerol (POL); 1,2-Dioleyl-3-palmitoleylglycerol (OOPo); 1-Palmitoyl-2,3-dilinoleylglycerol (PLL); 1-Palmitoleyl-2-oleyl-3-linoleylglycerol (PoOL); 1,3-Distearoyl-2-oleylglycerol (SSO); 1-Stearoyl-2,3-dioleylglycerol (SOO); 1,2,3-Trioleylglycerol (OOO); 1-Oleyl-2,3-dilinoleyglycerol (OLL); 1,2-Dioleyl-3-linoleyglycerol (OOL); 1,2,3-Trilinoleylglycerol (LLL) and 1-Arachidil-2,3-dioleylglycerol (AOO).

**Table 4:** Oxidative stability, total phenols content and phenolic profile of the eleven monovarietal extra virgin olive oils investigated.

	Carboncella	Coratina	Frantoio	Leccino	Marzio	Maurino	Moraiolo	Piantone di Falerone	Pendolino	Rosciola	Sargano di Fermo
Oxidative stability (h)	24.3 ^cd^ ± 0.4	29.5 ^a^ ± 0.9	19.8 ^f^ ± 0.4	17.5 ^g^ ± 0.7	24.2 ^cd^ ± 0.5	21 ^ef^ ± 1.1	26.3 ^bc^ ± 0.4	22.3 ^ed^ ± 0.7	20.3 ^ef^ ± 0.6	27.8 ^ab^ ± 0.8	19.6 ^f^ ± 0.6
Total phenols (mg gallic acid kg^–1^ oil)	323 ^b^ ± 10	283 ^cd^ ± 5.6	209 ^f^ ± 5.2	153 ^g^ ± 5.9	396 ^a^ ± 20	272 ^d^ ± 8.2	243 ^e^ ± 5.2	228 ^ef^ ± 10	308 ^bc^ ± 5.7	292 ^cd^ ± 9.4	208 ^f^ ± 6.5
**Phenolic compounds (mg kg^–1^ oil)**											
3,4-DHPEA	15.1 ^b^ ± 0.8	10.5 ^c^ ± 0.5	3.3 ^fg^ ± 0.1	18.5 ^a^ ± 1.4	6.1 ^e^ ± 0.1	4.9 ^ef^ ± 0.2	4.7 ^ef^ ± 0.1	8.2 ^d^ ± 0.3	1.8 ^g^ ± 0.1	15.7 ^b^ ± 0.9	4.5 ^ef^ ± 0.1
*p*-HPEA	3.1 ^ef^ ± 0.1	8.2 ^c^ ± 0.3	4.4 ^def^ ± 0.6	13.6 ^b^ ± 1.3	6.0 ^e^ ± 0.5	2.8 ^ef^ ± 0.2	2.2 ^f^ ± 0.3	5.1 ^de^ ± 0.3	4.3 ^def^ ± 0.5	16.8 ^a^ ± 1.1	3.5 ^def^ ± 0.5
Vanillic acid	0.29 ^c^ ± 0.3	0.52 ^c^ ± 0.1	1.1 ^a^ ± 0.1	0.92 ^b^ ± 0.1	0.82 ^b^ ± 0.1	1.34 ^a^ ± 0.2	nd	nd	0.49 ^c^ ± 0.1	0.63 ^bc^ ± 0.1	0.2 ^c^ ± 0.01
Vanillin	0.56 ^d^ ± 0.1	1.48 ^b^ ± 0.2	1.3 ^b^ ± 0.2	3.2 ^a^ ± 0.2	1.32 ^b^ ± 0.2	0.81 ^c^ ± 0.1	0.92 ^c^ ± 0.1	1.63 ^b^ ± 0.3	0.95 ^c^ ± 0.2	1.23 ^b^ ± 0.2	0.50 ^d^ ± 0.1
3,4-DHPEA-EDA	86.4 ^c^ ± 7.6	103 ^b^ ± 8.2	30.9 ^g^ ± 2.8	21.0 ^h^ ± 3.1	72.9 ^d^ ± 6.2	37.2 ^ef^ ± 2.1	80.1 ^cd^ ± 8.6	74.4 ^d^ ± 4.9	50.8 ^e^ ± 3.1	120 ^a^ ± 9.6	41.9 ^ef^ ± 3.9
*p*-HPEA-EDA	67.3 ^cd^ ± 5.5	64.4 ^cd^ ± 3.5	31.2 ^e^ ± 1.8	16.7 ^f^ ± 2.3	56.8 ^d^ ± 4.8	24.8 ^ef^ ± 2.8	82.3 ^b^ ± 6.8	71.8 ^bc^ ± 5.8	32.6 ^e^ ± 1.4	105 ^a^ ± 6.9	35.5 ^e^ ± 3.8
3,4-DHPEA-EA	53.5 ^cd^ ± 3.6	32.4 ^f^ ± 2.4	23.2 ^g^ ± 3.6	3.8 ^h^ ± 0.3	106 ^a^ ± 9.2	57.1 ^c^ ± 3.9	20.5 ^g^ ± 2.3	43.2 ^e^ ± 4.1	72.5 ^b^ ± 2.8	46.6 ^de^ ± 2.9	22.1 ^g^ ± 1.6
*p*-HPEA-EA	16.0 ^b^ ± 0.1	11.6 ^bc^ ± 1.8	14.7 ^b^ ± 0.7	2.9 ^d^ ± 1.2	23.3 ^a^ ± 2	11.5 ^bc^ ± 1.2	5.5 ^d^ ± 0.9	16.4 ^b^ ± 2.1	11.6 ^bc^ ±1.6	14.7 ^b^ ± 0.5	7.1 ^cd^ ± 1.1
Luteolin	8.3 ^a^ ± 1.1	5.45 ^c^ ± 0.5	6.9 ^b^ ± 2.3	nd	5.75 ^c^ ± 0.8	nd	5.45 ^c^ ± 1.1	2.65 ^d^ ± 0.3	4.25 ^c^ ± 0.6	nd	6.24 ^b^ ± 0.5
Apigenin	2.65 ^a^ ± 0.1	2.05 ^b^ ± 0.3	1.9 ^b^ ± 0.4	1.76 ^b^ ± 0.2	2.03 ^b^ ± 0.2	1.85 ^b^ ± 0.3	1.87 ^b^ ± 0.2	0.95 ^c^ ± 0.2	0.84 ^c^ ± 0.1	3.00 ^a^ ± 0.4	2.08 ^b^ ± 0.1

Oxidative stability (hours, induction time in response to force oxidation); phenolic substances abbreviation: 3,4-DHPEA: 3,4-Dihydroxyphenylethanol; *p*-HPEA: *p*-hydroxyphenylethanol; 3,4-DHPEA-EDA: dialdehydic form of decarboxymethyl elenolic acid linked to 3,4-DHPEA; *p*-HPEA-EDA: dialdehydic form of decarboxymethyl elenolic acid linked to *p*-HPEA; *p*-HPEA-EDA-Ox.: *p*-HPEA-EDA oxidized; 3,4-DHPEA-EA: oleuropein aglycon; *p*-HPEA-EA: ligstroside aglycon. Different letters (between a and h) in the same row indicate significantly different values (*p* < 0.001).
